# Carotid sinus hypersensitivity: block of the sternocleidomastoid muscle does not affect responses to carotid sinus massage in healthy young adults

**DOI:** 10.14814/phy2.13448

**Published:** 2017-10-16

**Authors:** Matthew G. Lloyd, James M. Wakeling, Michael S. Koehle, Robert J. Drapala, Victoria E. Claydon

**Affiliations:** ^1^ Department of Biomedical Physiology and Kinesiology Simon Fraser University Burnaby British Columbia Canada; ^2^ School of Kinesiology University of British Columbia Vancouver British Columbia Canada; ^3^ Division of Sport and Exercise Medicine University of British Columbia Vancouver British Columbia Canada

**Keywords:** Arterial baroreflex, carotid sinus hypersensitivity, sternocleidomastoid

## Abstract

The arterial baroreflex is crucial for short‐term blood pressure control – abnormal baroreflex function predisposes to syncope and falling. Hypersensitive responses to carotid baroreflex stimulation using carotid sinus massage (CSM) are common in older adults and may be associated with syncope. The pathophysiology of this hypersensitivity is unknown, but chronic denervation of the sternocleidomastoid muscles is common in elderly patients with carotid sinus hypersensitivity (CSH), and is proposed to interfere with normal integration of afferent firing from the carotid baroreceptors with proprioceptive feedback from the sternocleidomastoids, producing large responses to CSM. We hypothesized that simulation of sternocleidomastoid “denervation” using pharmacological blockade would increase cardiovascular responses to CSM. Thirteen participants received supine and tilted CSM prior to intramuscular injections (6–8 mL distributed over four sites) of 2% lidocaine hydrochloride, and 0.9% saline (placebo) in contralateral sternocleidomastoid muscles. Muscle activation was recorded with electromyography (EMG) during maximal unilateral sternocleidomastoid contraction both pre‐ and postinjection. Supine and tilted CSM were repeated following injections and responses compared to preinjection. Following lidocaine injection, the muscle activation fell to 23 ± 0.04% of the preinjection value (*P* < 0.001), confirming neural block of the sternocleidomastoid muscles. Cardiac (RRI, RR interval), forearm vascular resistance (FVR), and systolic arterial pressure (SAP) responses to CSM did not increase after lidocaine injection in either supine or tilted positions (supine: ΔRRI −72 ± 31 ms, ΔSAP +2 ± 1 mmHg, ΔFVR +4 ± 4%; tilted: ΔRRI −20 ± 13 ms, ΔSAP +2 ± 2 mmHg, ΔFVR +2 ± 4%; all *P* > 0.05). Neural block of the sternocleidomastoid muscles does not increase cardiovascular responses to CSM. The pathophysiology of CSH remains unknown.

## Introduction

The population is aging rapidly, with 9% (660 million people) aged >65 years worldwide, and this figure is expected to continue to rise substantially (Statistics Canada, 2010, 2014; United States Census Bureau, 2017). The risk of syncope is high in this age group (Moya et al. 2009), with a threefold higher risk of syncope in adults aged 70–79 years than adults aged 40–49 years (Ruwald et al. 2012). This increase likely contributes to falls and fall‐related injuries in older adults (Jansen et al. 2016). The impact of syncope and associated falls in older adults is severe ‐ elderly inpatients who faint display a 13–33% mortality in the first year and a 79% 4‐year mortality (Ungar et al. 2013). While the pathophysiology of syncope in the elderly is complex and incompletely understood, abnormal baroreflex function, particularly during postural change, has been implicated (Solbiati et al. 2016). Stretch receptors in the carotid arteries are central to the baroreflex response to changes in blood pressure during changes in body position. Up to 35% of healthy older adults and 26–60% of elderly patients with syncope display abnormally large responses when the carotid baroreceptors are stimulated using carotid sinus massage (CSM), a simple clinical test whereby manual massage of the skin overlying the carotid sinus is proposed to stimulate the underlying arterial baroreceptors (Moya et al. 2009). The high prevalence of CSH in the elderly, and its reported association with syncope (Moya et al. 2009), makes it an obvious candidate for consideration with respect to the increased risk of syncope and falling in older adults.

Despite its high prevalence in the elderly, the pathophysiology of CSH remains unknown, and several possible mechanisms have been proposed (Amin and Pavri 2015). One leading idea postulates that, in healthy individuals, stimulation of the sternocleidomastoid muscles near the carotid baroreceptors (during neck turning or carotid sinus massage), as well as the carotid baroreceptors themselves, leads to central integration of the two signals as “external” stretching of the sinus, and thus does not elicit a baroreflex response (Tea et al. 1996). Loss of this central integration due to sternocleidomastoid denervation might lead to hypersensitive responses during daily activities, such as neck turning, as well as during CSM (Blanc et al. 1997). This led to the first description of sternocleidomastoid electromyographic abnormalities in elderly patients with CSH (Blanc et al. 1997). It was suggested that chronic denervation of the sternocleidomastoid muscles with aging interrupts normal integration of neck muscle proprioception and carotid baroreceptor information, leading to excessive responses to CSM and an increased risk of syncope. We aimed to test this theory, hypothesizing that pharmacological block of the sternocleidomastoid muscles in healthy individuals will interrupt central integration of proprioceptive and carotid baroreceptor information, and lead to hypersensitive responses to CSM.

## Methods

### Ethical approval

This study was approved by the Department Of Research Ethics at Simon Fraser University and conforms to the principles outlined in the Declaration of Helsinki (World Medical Association, 2013). Prior to testing, participants provided written informed consent, and completed a brief medical history to confirm they met our inclusion criteria. Participants were excluded from the study if any of the following criteria were met: pregnancy; history of neck surgery, neck injury, or currently symptomatic neck pain; known sensitivities or allergies to medications containing lidocaine; pre‐existing cardiovascular or neurological disease; use of medications containing lidocaine or with cardiovascular actions.

### Protocol

The carotid arteries were screened for the presence of stenosis using ultrasound, and the ultrasound‐guided location of the carotid bifurcation was determined and marked on the skin. After a 10‐min supine rest period, participants then underwent first supine and then tilted CSM in duplicate, on both sides of the neck. Muscle activation (electromyography, EMG) of the sternocleidomastoid muscles during standardized maximum voluntary contraction was recorded. Participants then received intramuscular injections using a 25G 1” needle (8 mL distributed over four sites) of 2% lidocaine hydrochloride, and 0.9% saline (placebo) in contralateral sternocleidomastoid muscles (determined randomly). After a 10‐min rest period (to allow time for optimal block from the lidocaine injections and the recovery of any cardiovascular responses to any perceived discomfort acutely associated with the injections) EMG recordings were repeated following injection to quantify the extent of block of muscle activity with lidocaine. Supine and tilted CSM were then repeated to determine the impact on cardiovascular responses obtained. Data were collected and analyzed in a double‐blind fashion.

### Screening for carotid stenosis

Participants' carotid arteries were screened for the presence of significant stenosis, and participants were excluded from further testing if arterial narrowing >50% was detected by the study physician. Examinations were conducted using the GE Logiq I system (GE Healthcare, Chicago, IL), with a 6.3 MHz linear transducer. The carotid arteries on both sides were evaluated in both transverse and longitudinal planes for the presence of visible significant narrowing on B‐mode grayscale image and the presence of mosaic pattern on color Doppler image. Examinations were performed with the standard presets for carotid ultrasound initially, with technician optimization where necessary. The location of the carotid bifurcation was identified and marked on the skin.

### Cardiovascular monitoring

Beat‐to‐beat cardiac (R‐R interval [RRI]: electrocardiography [ECG]; Lead II) and blood pressure responses to CSM were recorded using a Finometer Pro (Finapres Medical Systems BV, Amsterdam, the Netherlands). Finger cuff measurements were calibrated to brachial blood pressure using the return‐to‐flow calibration (Guelen et al. 2008) prior to commencing testing, and throughout using the internal calibration (Physiocal™). Immediately prior to CSM, the Physiocal™ was turned off to prevent interruption of the waveform. The participants' hand was kept at approximately heart height throughout testing, and a height correction unit was used to account for small changes in vertical height between the finger cuff and the heart (Guelen et al. 2008). Brachial artery blood flow velocity was measured using an 8 MHz probe positioned overlying the brachial artery and clamped at a constant angle throughout testing (Doppler‐Box™, Compumedics, DWL, Singen, Germany). Forearm vascular resistance was calculated as mean arterial pressure/brachial blood flow velocity.

### Carotid sinus massage

**Figure 1:**
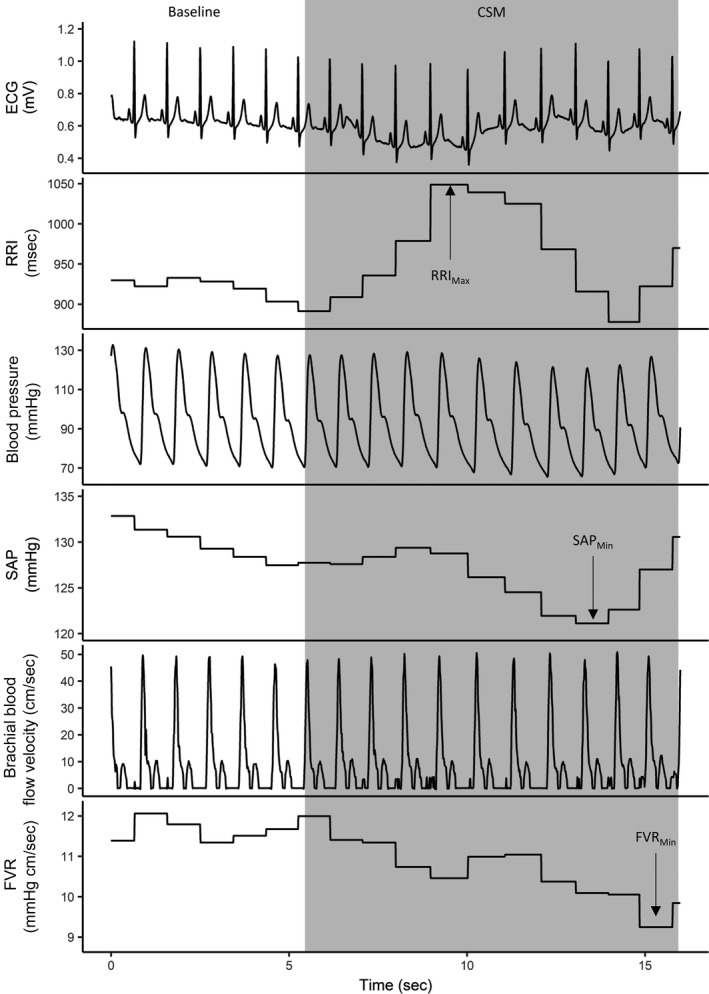
A representative example tracing from one individual showing cardiac, blood pressure, and forearm vascular resistance responses to carotid sinus massage. The period of massage is indicated by the grey box. Note the modest bradycardia and vasodilation induced by the massage. Responses were calculated as the appropriate minimum (Min) or maximum (Max) value (indicated) compared to the mean value for 5‐sec prior to the onset of massage (baseline). CSM, carotid sinus massage; ECG, electrocardiogram; RRI, RR interval; SAP, systolic arterial pressure; FVR, forearm vascular resistance.

Carotid sinus massage was performed for 10 sec at the ultrasound‐guided location of the carotid bifurcation. Participants were instructed to hold their breath at the end of normal expiration for 15 sec; CSM occurred during the last 10 sec of the breath‐hold in order to minimize the potential confounding influence of respiration on the responses obtained. Two CSM were performed and responses averaged on each side of the neck with the participant supine, and again during 70‐degree head‐up tilt. The pressure and technique of the massage stimulus was standardized by having the same investigator perform all massages. A representative example tracing showing responses to CSM can be seen in Figure 1.

### Muscle activation

Muscle activation was measured with EMG. Surface EMG signals (bipolar Na/NaCl electrodes) were recorded from the sternocleidomastoid muscles halfway between the mastoid process and sternum. Signals were recorded at a frequency of 2000 Hz and amplified with a bandwidth of 10–500 Hz (Biovision, Wehrheim, Germany). Prior to electrode placement the area was shaved, abraded and the skin cleaned with alcohol. Maximal unilateral sternocleidomastoid contraction was performed with the participant supine, and the head rotated. Participants were instructed to lift their head off the table and maximally push against a standardized opposing force for 7‐sec, with EMG recorded during the last 5‐sec of contraction. Two repetitions were performed on each side.

### Data analyses

Data recorded from the side of the neck that received lidocaine injection were considered “lidocaine” and from the side that received saline injection were considered “placebo”. Data were recorded with a sampling frequency of 1KHz (Powerlab 16/30, AD Instruments, Colorado Springs, CO), acquired using LabChart (AD Instruments) and stored for offline analysis. ECG and brachial blood flow velocity signals were filtered using a 50 Hz low‐pass filter. Brachial blood flow velocity was calculated as the area under the curve of the filtered signal. Cardiovascular responses to CSM were calculated as the minimum or maximum value (as directionally appropriate for the known physiology) during massage compared to the mean value for 5‐sec prior to the onset of massage. RRI responses were also determined as the longest absolute RRI during the CSM. Responses of the two repetitions for each side were averaged within each condition (lidocaine and placebo) and position (supine and tilt). Responses to CSM were classified as hypersensitive if there was an asystole lasting >3 sec, and/or a fall in systolic blood pressure >50 mmHg (Moya et al. 2009). A representative example tracing showing responses to CSM can be seen in Figure 1. Sternocleidomastoid muscle activity was quantified as the root‐mean‐square (RMS) of the EMG signals from the last 5 sec of each contraction. The postinjection RMS EMG was normalized to the maximum preinjection levels. RMS EMG for the two postinjection contractions on one side were averaged and expressed relative to the baseline.

## Statistics

Data were analyzed using R (3.2.3), Rstudio (0.99.902) and SigmaPlot Version 12. Paired *T*‐tests were used to compare changes in EMG activity with placebo and lidocaine injections. Repeated‐measures ANOVA with factors for treatment (lidocaine vs. placebo), condition (supine vs. upright) and time (preinjection vs. postinjection) were used to compare cardiovascular responses. Residual versus fitted plots and q‐q plots were generated to confirm normality of the residuals, and standard deviations of each group were compared to confirm the assumption of equal variance. Holm‐Sidak post hoc tests were used to analyze interaction terms, with *P* < 0.05 set as the cut‐off for significance. Data are represented as mean ± standard error unless otherwise stated.

## Results

Thirteen participants (aged 28 ± 1 years; height 173 ± 2 cm; weight 71 ± 3 kg; eight male) were recruited for the study. One participant experienced a mild vasovagal reaction to the injections, and completed only two CSM during head‐tilt following the injections, one on each side of the neck.

### Muscle activity

**Figure 2:**
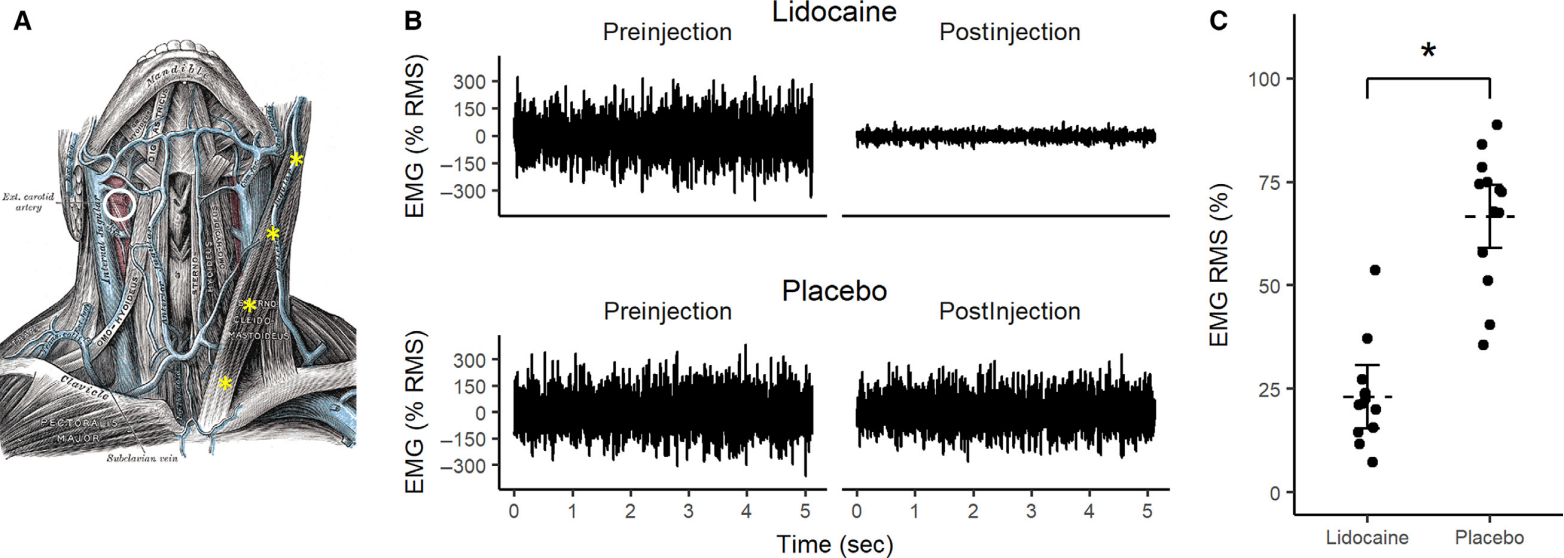
Effect of lidocaine administration on sternocleidomastoid electromyography (EMG). (A) Drawing showing the anatomical locations of the key structures in the neck. Carotid sinus massage was performed at the location of the carotid bifurcation determined using ultrasound (white circle). Lidocaine or placebo injections (2 mL per site) were conducted on contralateral sternocleidomastoid muscles at four injection sites encompassing the full length of the muscle (yellow stars). (B) Representative EMG recorded from one participant before and after lidocaine and placebo injections. Traces show EMG recordings for the last 5 sec of a 7‐sec maximal voluntary contraction. There was a near‐abolition in EMG following lidocaine injection. EMG is expressed as percent of maximum root mean squared (RMS) during preinjection contraction. (C) Group data showing RMS EMG after injection with lidocaine and placebo (expressed as percent of maximum preinjection contractions). The horizontal dashed lines represent group means, with solid horizontal lines representing the 95% confidence interval; *Denotes statistical significance (*P* < 0.0001).

Following injection of lidocaine the muscle (RMS) activity fell to 23 ± 0.04% of the maximum preinjection level, compared to 67 ± 0.05% for placebo (*P* < 0.0001, Fig. 2).

### Carotid sinus massage

Baseline responses to CSM prior to injection were small and not significantly different between placebo or lidocaine conditions in either the supine or tilted position (Table 1). Resting supine RRI, SAP and FVR prior to CSM were not significantly different between pre‐ and postinjection conditions.

**Table 1:** Cardiovascular responses to placebo and lidocaine injection in the supine and tilted conditions

	Supine				HUT			
	Preinjection		Postinjection		Preinjection		Postinjection	
	Lidocaine	Placebo	Lidocaine	Placebo	Lidocaine	Placebo	Lidocaine	Placebo
Max RRI (ms)	1075 ± 33	1090 ± 40	1030 ± 44	1023 ± 50	864 ± 49*	895 ± 53*	840 ± 48*	859 ± 47*
Δ RRI (ms)	+111 ± 37	+103 ± 32	+41 ± 9^†^	+39 ± 14^†^	+67 ± 12	+104 ± 25	+67 ± 10	+64 ± 16
Δ SAP (mmHg)	−5 ± 1	−7 ± 1	−3 ± 1	−5 ± 1	−5 ± 1	−6 ± 2	−3 ± 1	−6 ± 2
Δ FVR (%)	−18 ± 3	−23 ± 3	−16 ± 4	−16 ± 3	−15 ± 4	−16 ± 4	−13 ± 4	−15 ± 4

Responses are expressed as the largest response during carotid sinus massage, relative to the pre‐massage baseline. RRI, RR interval; SAP, systolic arterial pressure; FVR, forearm vascular resistance; HUT, head‐upright tilting. Significant differences (*P* < 0.05) between corresponding values during supine and HUT are denoted by *; significant (*P* < 0.05) main effect of injection in the supine position (postinjection vs. preinjection, independent of lidocaine or placebo condition) is denoted by †.

The maximum RRI during CSM while supine was greater than while tilted in both placebo and lidocaine conditions, pre‐ and postinjection (*P* < 0.001). However, the change in RRI (maximum prolongation during CSM compared to the mean RRI during the 5‐sec before CSM) was not significantly different between supine and tilt conditions either pre‐ or postinjection, for either placebo or lidocaine. The changes in SAP and FVR were also not significantly different between supine and tilt conditions either pre‐ or postinjection, for either placebo or lidocaine.

In both placebo and lidocaine conditions, there was a significant main effect of injection independent of receipt of placebo or lidocaine injections, whereby the change in RRI during the supine phase was *smaller* postinjection than preinjection (*P* < 0.05). However, this was not statistically significant with post hoc comparisons within each injection condition (*P* = 0.07 for both placebo and lidocaine conditions, respectively). There was no effect of injection on responses during tilted conditions.

**Figure 3:**
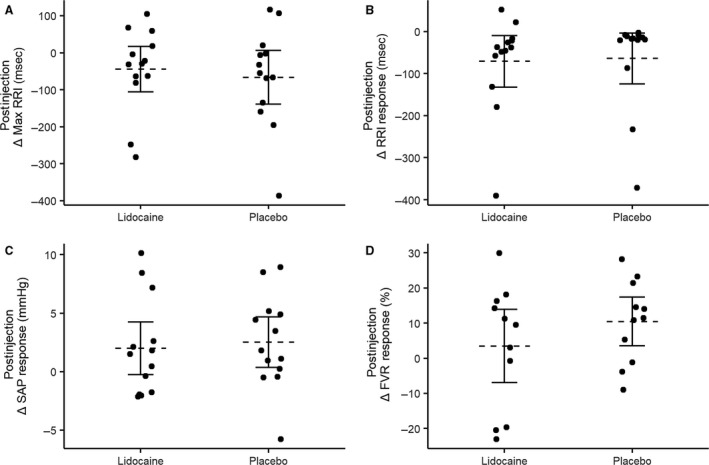
Effect of lidocaine and placebo injection on the supine cardiovascular responses to carotid sinus massage (CSM). Responses are expressed as the change in response to CSM in the postinjection condition compared to the preinjection condition for: (A) maximum RRI prolongation; (B) change in RRI; (C) change in SAP; and (D) change in FVR. Horizontal dashed lines represent group means, solid horizontal lines represent 95% confidence interval. RRI, RR interval; SAP, systolic arterial pressure; FVR, forearm vascular resistance.

**Figure 4:**
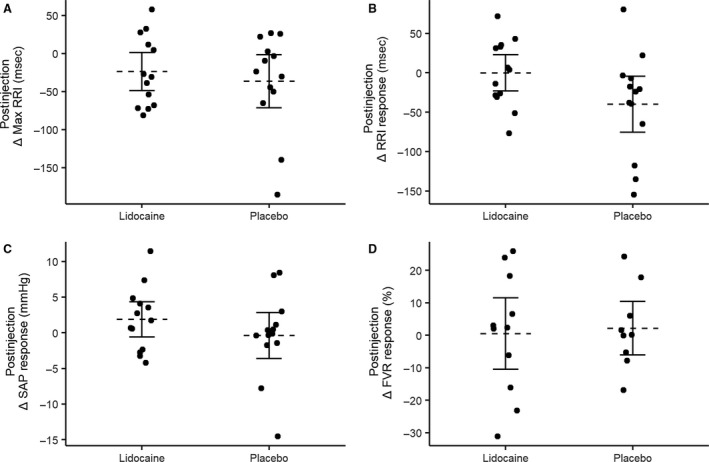
Effect of lidocaine and placebo injection on the tilted cardiovascular responses to carotid sinus massage (CSM). Responses are expressed as the change in response to CSM in the postinjection condition compared to the preinjection condition for: (A) maximum RRI prolongation; (B) change in RRI; (C) change in SAP; and (D) change in FVR. Horizontal dashed lines represent group means, solid horizontal lines represent 95% confidence interval. RRI, RR interval; SAP, systolic arterial pressure; FVR, forearm vascular resistance.

Our primary interest was in whether the responses to CSM were increased following injection of lidocaine. Therefore, we expressed the cardiovascular responses to CSM postinjection relative to preinjection for both the lidocaine and placebo drug administration during supine (Fig. 3) and tilted conditions (Fig. 4). The postinjection changes in cardiovascular responses were not significantly different from zero in either supine (Fig. 3) or tilted (Fig. 4) positions, during either placebo or lidocaine conditions; there was no effect of injection of lidocaine or placebo on cardiovascular responses to carotid sinus massage.

Hypersensitive responses were not observed in any participant in any condition.

## Discussion

We showed that neural block of the sternocleidomastoid muscles does not increase cardiovascular responses to CSM in healthy young controls. This is contrary to the hypothesis (Blanc et al. 1997) that chronic denervation of the sternocleidomastoid muscles with aging interrupts normal integration of neck muscle proprioception and carotid baroreceptor information, leading to excessive responses to CSM and an increased risk of syncope. Our results suggest that the previously reported association between sternocleidomastoid denervation and CSH in older adults (Blanc et al. 1997) may be coincidental, rather than causal, and highlight the need for further research to identify the pathophysiological mechanisms underlying CSH.

We are confident that we achieved an effective block of the sternocleidomastoid muscles with lidocaine based on the known short onset of action (3–5 min) and relatively long half‐life (1.5–2 h) of lidocaine, as well as the near‐abolition of sternocleidomastoid muscle activity that we observed following injection. Given that afferent fibers are blocked with lower doses of lidocaine than motor fibers (Garmon and Dulebohn 2017), we consider the afferent input from the muscle to have been lost during the lidocaine condition; therefore, we infer that our negative findings are not a consequence of inadequate block.

Rather than the proposed enhancement of cardiovascular responses to CSM, we actually saw a small but statistically significant *decrease* in RRI responses to CSM following injection. This small reduction was independent of the placebo or lidocaine conditions and may reflect the impact of the volume of injected fluid within the muscle on massage stimulus transmission to the carotid sinus.

In healthy young adults, we observed larger cardiac responses to CSM when performed supine than when tilted, independent of the lidocaine or placebo condition. This is of interest because the current clinical guidelines (Shen et al. 2017) suggest that tilted CSM may be of benefit in patients with recurrent syncope and/or suspected CSH because of the potential to unmask pathologically large vasodepressor responses in susceptible individuals when CSM is combined with orthostatic stress. This was not the case in our studies, presumably because the baroreflex engagement associated with normal orthostatic control in young healthy individuals (and resultant increases in sympathetic stimulation of the heart and vasculature) blunts the magnitude of the evoked bradycardia and vasodilation during CSM. If this observation extends to older adults, and those with CSH, it would suggest that orthostatic CSM may not be a necessary addition to autonomic function testing in these individuals.

Our results do not support the prior observation (Blanc et al. 1997) of sternocleidomastoid denervation contributing to CSH in older adults. This discrepancy may partly reflect the different methodology employed in the previous study, whereby sternocleidomastoid raw EMG was qualified based on physician observation as “normal” or “abnormal – moderate or severe,” and not subjected to quantitative evaluation. The method of standardization of the stimulus for contraction of the sternocleidomastoid muscles was also not reported in the previous study so it is unclear whether abnormal EMG could be influenced by reduced contractile effort. Similarly, the CSM stimulus in the previous study was not standardized, and again responses were qualified as “normal, doubtful or CSH.” Where responses were discrepant between sides or there was disconnect between cardiac and blood pressure responses to CSM, the most abnormal response was considered in the correlative analyses. Nevertheless, despite these methodological differences, in the previous study abnormal EMG were observed in 57% of individuals and CSH in 27% of individuals, with strong concordance (*P* < 0.00001) between the two (Blanc et al. 1997). However, correlation does not necessarily imply causality. It may be that these results reflect the known associations between aging and sarcopenia and age‐related muscle weakness (reflected in reduced EMG activity) (Clark and Fielding 2012), and between aging and carotid sinus hypersensitivity (Moya et al. 2009), rather than an interaction between sternocleidomastoid muscle denervation and CSH per se.

We have refuted the proposed potential role of chronic sternocleidomastoid denervation in the pathophysiology of CSH. While this adds an interesting new piece to this physiological puzzle, the mechanism underlying CSH remains elusive. Currently, two additional hypotheses have attempted to explain the phenomenon of CSH. The first posits that with aging, atherosclerotic buildup on the carotid artery vessel walls results in increased vessel stiffness, reduced barororeceptor activity, and upregulation of central gain with consequent overshoot responses (O'Mahony 1995). While patients with CSH do exhibit increased arterial stiffening (Madden et al. 2010), efforts to investigate the mechanism of increased central gain have been unsuccessful (Kenny et al. 1987; Parry et al. 2004).

The second hypothesis argues that CSH is a symptom of more widespread autonomic dysregulation, including increased baroreflex gain and increased resting sympathetic tone (Tan et al. 2010). The pathophysiology of this dysregulation is unknown, but may involve altered function of central pathways involved in the baroreflex. For example, CSH is common in patients with dementia with Lewy bodies, which points to central white matter lesions as contributors to the large heart rate and systolic blood pressure responses to CSM (Kenny et al. 2004). In patients with CSH, increased tau accumulation in baroreflex‐associated nuclei has been observed (Miller et al. 2008), but cause and effect in terms of CSH has yet to be demonstrated.

## Limitations

We saw a small but statistically significant reduction in the EMG signals following the placebo injections. We assume this reflects either modest fatigue during repeated EMG testing, or the presence of the volume of the injected saline within the muscle belly either slightly impairing contraction or providing impedance to the EMG signals recorded. However, we do not believe this influences our overarching conclusion – the small reduction in EMG signals in the placebo condition was far less than the near‐abolition of the EMG signals with lidocaine injections. Furthermore, the main implication of the small decrease in EMG activity in the saline condition would be hypothesized to be a small enhancement of the responses to CSM; this was not the case, further supporting our findings that neural block of the sternocleidomastoid muscles does not increase cardiovascular responses to CSM.

We had a relatively small sample size and of course this has the potential to influence the statistical power. However, perhaps in part because of the highly standardized conditions, (ultrasound‐guided carotid sinus massage; standardized massage procedure and pressure applied; breath holding to exclude respiratory sinus arrhythmia and respiratory‐induced changes in blood pressure) we had very low within‐subject variance. Accordingly, we were powered to detect a difference in the RRI response following injection of just 60 ms, and for blood pressure and vascular responses of only 8 mmHg and 7%, respectively. We do not think the lack of enhancement of cardiovascular responses to CSM with sternocleidomastoid blockade can be explained by low statistical power.

We conducted our analyses in a cohort of young healthy participants, who had small baseline responses to CSM with no evidence of hypersensitive responses; this phenomenon is well‐documented (Krediet et al. 2011). We chose to conduct our assessments in young individuals with small responses to CSM because we were concerned that the larger responses documented in older adults (Moya et al. 2009; Humm and Mathias 2010; Krediet et al. 2011; Sutton 2014) might create a ceiling effect, and that it would not be possible to enhance responses further with the lidocaine intervention in this population, leading to a “false‐negative” result. Accordingly, we are confident that the lack of increase in response to CSM during lidocaine administration was not due to a ceiling effect. However, given the known effect of aging on baroreflex responses to CSM, it is possible that responses in healthy older adults to the intervention might be different (smaller) than those reported here in young individuals.

Finally, we considered the potential role of only the sternocleidomastoid muscles in the pathophysiology of CSH, and did not consider that normal afferent feedback from other neck muscles might compensate for the imposed pharmacological “denervation” of the sternocleidomastoid muscle in this study. We do not believe this invalidates our findings for two reasons: firstly, we did not provide an afferent stimulus to any other muscle groups during our CSM protocol so they presumably did not ameliorate the impact of our intervention; secondly, prior research has shown that only reductions in EMG of the sternocleidomastoid muscles, and not other neck muscles (such as styloglossus and upper trapezius) were associated with CSH (Tea et al. 1996).

## Conflict of Interest

The authors have no conflicts of interest to report.
